# An Integrative Analysis Identified Six Genes That Regulate the Development of Atherosclerosis Through Autophagy

**DOI:** 10.1155/ijog/9962311

**Published:** 2025-09-23

**Authors:** Ao Yin, Xingyu Fu, Xinxin Liu, Min Li, Xiaochen Yu, Xiuru Guan

**Affiliations:** ^1^ Department of Laboratory Medicine, First Affiliated Hospital of Harbin Medical University, Harbin, Heilongjiang, China, hrbmu.edu.cn

**Keywords:** atherosclerotic, autophagy, bioinformatics analysis, consensus clustering analysis, machine learning, quantitative real-time PCR

## Abstract

**Background:** Autophagy exerts a vital role in the development of atherosclerotic lesions. Mounting evidence suggests a significant link between autophagy and atherosclerosis.

**Methods:** Two atherosclerotic plaque datasets were integrated from the Gene Expression Omnibus (GEO) database. After differentially expressed genes (DEGs) were determined, enrichment analyses were subsequently performed on DEGs. We employed weighted gene coexpression network analysis (WGCNA) and cross‐linked these modules with DEGs and autophagy‐related genes. Subsequently, a prediction model was established for evaluation. RT‐PCR was adopted to identify hub gene expression. The consensus clustering analysis on the overlapping genes was executed. Evaluation of immune infiltration was conducted on the merged dataset. A TF‐miRNA‐mRNA regulatory network was then established for the hub genes.

**Results:** The differential gene expression analysis uncovered 259 DEGs. Enrichment analysis showed that immune and inflammatory reactions were related to atherosclerosis. By intersecting DEGs, WGCNA module genes, and ARGs, 13 overlapping genes were obtained. Four machine learning models identified seven hub genes. Furthermore, six of the seven genes demonstrated potential for disease diagnosis. The prediction model, based on the expression levels of these six genes, yielded satisfactory results. RT‐PCR analysis demonstrated that the mRNA expression of six genes meets expectations. Consensus clustering divides 13 overlapping genes into two clusters, C1 and C2, with significant differences in immune infiltration. Immune cell infiltration demonstrated heightened immune activity within the atherosclerotic plaque group. A TF‐miRNA‐mRNA regulatory network was established for the six genes.

**Conclusion:** It is anticipated that these six genes may serve as significant and valuable targets for future research into atherosclerosis.

## 1. Introduction

Atherosclerosis, characterized by chronic inflammation, is the principal factor triggering cardiovascular diseases. The pathogenesis of atherosclerosis involves the interplay of multiple regulatory factors, including disturbances in glucose and lipid metabolism, endothelial injury, and inflammatory responses [1]. Autophagy, a key cellular mechanism, is essential for maintaining homeostasis by metabolizing intracellular components and renewing specific organelles. In the autophagic process, damaged proteins or organelles are encapsulated by double‐membrane autophagosomes and transported to lysosomes or vacuoles for degradation and recycling. A wide spectrum of human diseases, such as atherosclerosis, is associated with impairments in autophagy [2]. Enhanced autophagy might improve the stability of atherosclerotic plaques, thus contributing to the therapeutic management of atherosclerosis [3]. Autophagy‐related genes (ARGs) play a crucial role in this process, with both their overexpression and underexpression being closely linked to atherosclerosis. For instance, the deletion of ULK1 impairs autophagy and inhibits vascular smooth muscle cell (VSMC) migration [4], while reduced expression of BECN1 attenuates endothelial cell autophagy and diminishes protection against atherosclerosis [5]. Conversely, enhanced expression of ARG2 suppresses autophagy, which is also associated with atherosclerosis [6].

With the rapid progress of bioinformatics in recent years, high‐throughput sequencing data, microarray data, and single‐cell sequencing data, among other bioinformatics approaches, have become essential for modern medical research. Among these, the Gene Expression Omnibus (GEO) database is commonly employed for data mining to uncover metabolic pathways and therapeutic targets in nononcological conditions.

**Figure 1:**
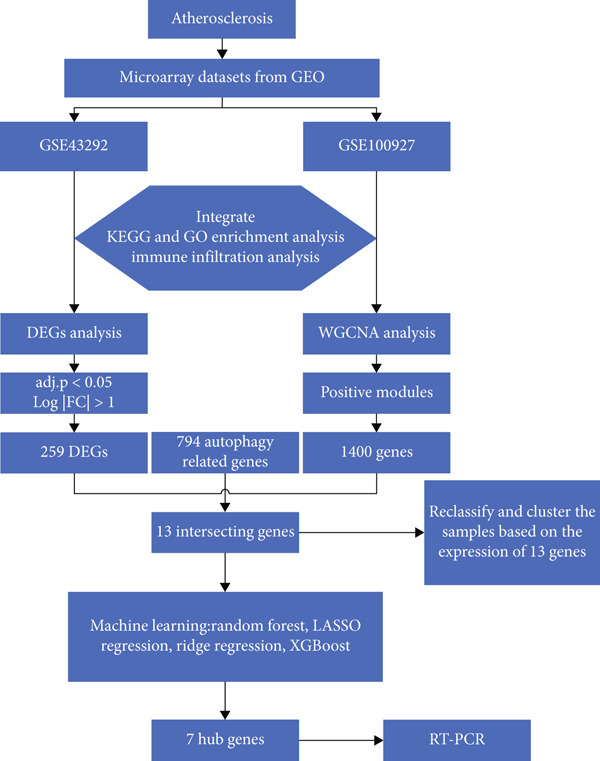
Flowchart of integrative analysis.

By analyzing the atherosclerosis plaque‐related datasets GSE43292 and GSE100927 in GEO datasets and using a variety of bioinformatics methods, this study was aimed at examining differentially expressed autophagy‐related genes (DEARGs) in atherosclerotic plaques and employing machine learning to identify hub genes. Ultimately, an atherosclerosis model was created by exposing human macrophages to oxidized low‐density lipoprotein (ox‐LDL), and the expression of these genes was validated in atherosclerosis. The workflow is illustrated in Figure 1.

## 2. Materials and Methods

### 2.1. Data Acquisition and Preprocessing

Two gene expression matrices (GSE43292 and GSE100927) were acquired from the GEO database (http://www.ncbi.nlm.nih.gov/geo) for this study. GSE43292, which utilized the GPL6244 platform, provided expression data for 32 atherosclerotic plaque samples and 32 normal tissue controls. Utilization of the GPL17077 platform in the GSE100927 dataset included 72 samples of atherosclerotic plaque and 35 samples of normal artery tissue. Subsequently, the GSE100927 and GSE43292 datasets were pooled to create a new dataset that was used for downstream analysis. Following this, batch effects were addressed using the SVA package. The criterion for statistical significance was set at a *p* value less than 0.05. We set the newly merged dataset as the training set. Principal component analysis (PCA) was utilized to visualize the batch effects within the combined expression matrix. The GSE57691 dataset of the GPL10558 platform was used as the verification set, containing nine atherosclerotic samples and 10 normal samples. We found 794 ARGs in the human autophagy regulatory database (http://hamdb.scbdd.com/home/index/) (Table S1).

### 2.2. Screening of Differentially Expressed Genes (DEGs)

The “limma” R package was employed to analyze the new dataset and identify DEGs linked to atherosclerosis. Genes with an adjusted *p* value < 0.05 and a fold change (FC) > 2 were categorized as DEGs [7]. The identified DEGs were then visualized through a volcano plot generated by the Sangerbox platform (http://vip.sangerbox.com/home.html) [8].

### 2.3. Functional Enrichment Analysis

Functional interactions of the DEGs were explored using Gene Ontology (GO) and KEGG enrichment analyses, performed utilizing the clusterProfiler package in R. Target gene functions were annotated using the GO system according to biological process (BP), molecular function (MF), and cellular component (CC) [9]. KEGG pathway enrichment analysis was used to associate target genes with the biological pathways in which they were involved. A cutoff of 0.05 for the corrected *p* value was employed to assess statistical significance [10]. A bubble chart was chosen to present the findings of the analysis.

### 2.4. Weighted Gene Coexpression Network Analysis (WGCNA)

WGCNA is a robust bioinformatics methodology employed to discern modules of correlated genes and analyze the links between gene networks and significant phenotypes. The methodology also facilitates the identification of hub genes within these established networks. After eliminating outlier genes, the WGCNA package in R was utilized to generate a weighted gene coexpression network [11]. This package facilitated the identification of modules exhibiting the strongest association with atherosclerosis. Consequently, we identified 22 coexpression modules and subsequently extracted the genes from key modules.

### 2.5. Clustering of Atherosclerotic Samples

The consensus clustering analysis was carried out on the expression matrix of 13 overlapping genes utilizing the ConsensusClusterPlus R package [12]. The number of clusters (*k*) was explored, ranging from 2 to 10. Optimal clustering, characterized by the highest intracluster correlation and a comparatively low intercluster correlation, was achieved with *k* = 2. For the consensus clustering procedure, the maximum number of clusters was set to 6, with 10,000 resampling iterations and a sampling fraction of 0.8. As a result, a consensus clustering heat map, a cumulative distribution function (CDF) graph, and a delta area chart were generated.

### 2.6. Application of Machine Learning Algorithms for Gene Screening

To identify key atherosclerosis‐related ARGs from overlapping genes, we employed the randomForest and XGBoost packages to perform random forest importance scoring and XGBoost importance scoring on the overlapping genes [13, 14]. LASSO and ridge regression analyses on overlapping genes were implemented leveraging the glmnet package [15, 16]. Genes that were consistently identified across all four machine learning algorithms were considered hub genes associated with autophagy and atherosclerosis.

### 2.7. Construction of a Predictive Model for Autophagy–Atherosclerosis Genes

We analyzed the ROC curve of the genes screened by machine learning using the pROC package [17]. In the validation set, genes with an area under the ROC curve greater than 0.6 were useful for disease diagnosis. These screened genes were used to construct a logistic regression model. The accuracy of the prediction model and other results was visualized, and the ROC curve was plotted using the pROC package.

### 2.8. Immune Infiltration Analysis

To explore the correlation of immune infiltration with the pathogenesis of atherosclerosis, we utilized the CIBERSORT algorithm, implemented through the R package IOBR, to examine variations in the quantities of 22 immune cell types in atherosclerotic plaque and control samples. This analysis was performed on a newly integrated matrix. The total proportion of 22 immune cell types was constrained to 1 for each sample. Differences reaching a statistical significance threshold of *p* < 0.05 were considered significant [18] and were represented graphically with box plots.

### 2.9. Construction of TF‐MicroRNA (miRNA)‐mRNA Regulatory Network

Transcription factors are key regulators of target gene expression. TFs related to hub genes were identified utilizing the hTFtarget (http://bioinfo.life.hust.edu.cn/hTFtarget#!/) database. The database was curated with 7190 ChIP‐seq datasets for 659 TFs, drawn from ENCODE, GEO, and SRA. Additionally, it incorporated high‐confidence DNA‐binding sequences for 699 TFs, sourced from TRANSFAC, JASPAR, and HOCOMOCO [19]. This was aimed at determining the association between TF regulation and hub genes. miRNAs are key posttranscriptional regulators of gene expression, and their aberrant activity significantly contributes to the onset and progression of numerous diseases [20]. We employed the six identified hub genes as target genes and used the starBase database to identify miRNAs that regulate these genes (https://starbase.sysu.edu.cn/index.php) [21]. In the starBase database, predicted interactions were gathered from seven distinct databases, specifically TargetScan, picTar, RNA22, PITA, miRanda, miRmap, and microT. Interactions that were predicted by two or more databases were classified as high‐confidence. The regulatory network was visualized with Cytoscape.

### 2.10. Verification of the Expression of Hub Gene of Autophagy–Atherosclerosis in Foam Cells

Humanized THP‐1 cells were cultivated in RPMI‐1640 medium (bought from Shanghai Shangbao Biological Technology Co. Ltd) enriched with 10% fetal bovine serum (FBS). The culture medium was maintained at 37°C with 5% carbon dioxide (CO_2_). Cells were passaged at 80%–90% confluence using a 1:2 split ratio. A 48‐h treatment with 100 *μ*g/mL PMA was used to differentiate THP‐1 cells into macrophages. At this stage, THP‐1 cells have adhered and differentiated into macrophages. The medium containing PMA was then discarded and washed with PBS for 2–3 times. Then, macrophages were treated with 25 *μ*g/mL ox‐LDL (bought from Guangzhou Yiyuan Biotechnology Co. Ltd) for 48 h, resulting in foam cell formation. Subsequently, the cells were observed using an optical microscope.

### 2.11. Reverse‐Transcription Quantitative PCR

Macrophages and foam cells were harvested, and total RNA was extracted utilizing TRIzol reagent. Following RNA isolation, cDNA was synthesized through reverse transcription. Six key genes were then analyzed using real‐time qPCR to determine their expression levels. Gene expression levels were quantified utilizing the 2^−*Δ* *Δ*Ct^ technique. The PCR protocol began with an initial 30‐s denaturation at 95°C (1 cycle), which was then succeeded by 40 cycles of amplification. Each amplification cycle consisted of a 15‐s denaturation at 95°C and a 25‐s annealing and extension at 60°C. Table S2 provides primer information.

## 3. Results

### 3.1. Data Processing and DEG Screening and Analysis

PCA plots were used to visualize batch effects in the expression matrix derived from the GSE43292 and GSE100927 datasets (Figure 2a,b). The expression patterns of several samples from two distinct datasets are depicted in Figure 2. A comparison of Figure 1a,b demonstrated that the “sva” package effectively removed batch effects after integrating the two different datasets [22]. This indicated that the merged data demonstrated the requisite characteristics for further analysis and processing. GSE43292 comprised 32 nonatherosclerotic and 32 atherosclerotic plaque samples. Similarly, GSE100927 included 35 nonatherosclerotic and 72 atherosclerotic plaque samples. Subsequently, the “limma” package was employed for DEG analysis of the merged expression matrix, revealing 62 downregulated and 197 upregulated genes. The outcomes were presented in the form of a volcano plot (Figure 2c).

Figure 2(a) PCA score of GSE43292 and GSE100927 before integration. (b) PCA score of GSE43292 and GSE100927 after integration using limma. (c) Volcano plot of differentially expressed genes. Red data points represent upregulated genes, green data points represent downregulated genes, and differential screening multiple is 2, adjusted *p* value < 0.05.

**Figure:**
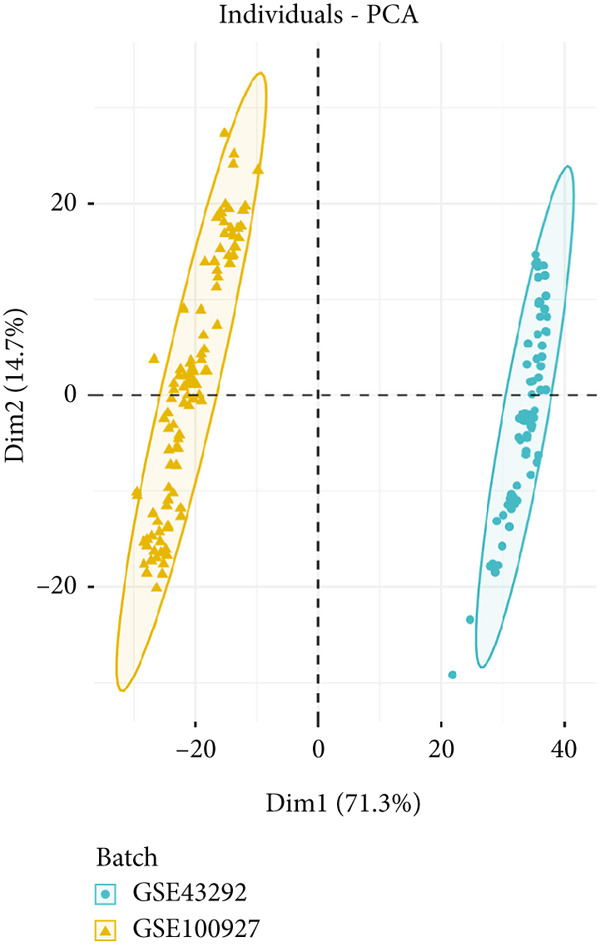
(a)

**Figure:**
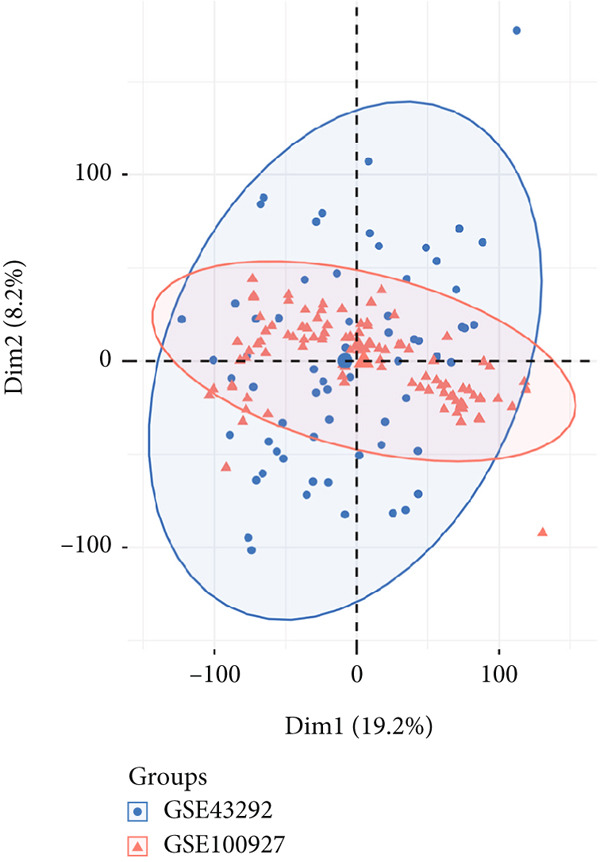
(b)

**Figure:**
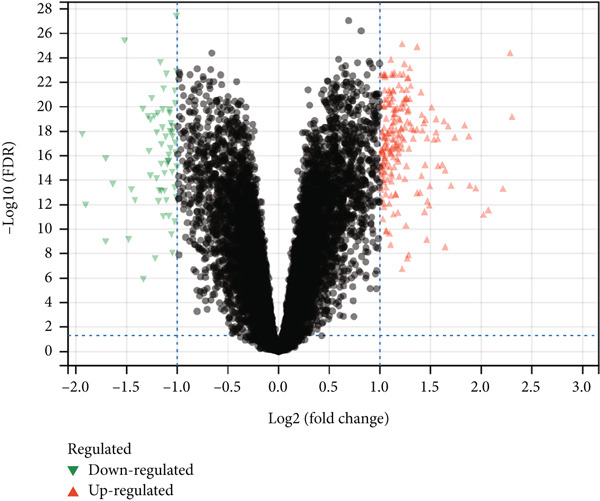
(c)

### 3.2. GO Functional Annotation and KEGG Signaling Pathway Enrichment Evaluation of DEGs

GO functional annotation and KEGG pathway enrichment evaluation were carried out on the DEGs utilizing the “clusterProfiler” package in R. Within the BP category, DEGs were significantly enriched in immune‐related pathways, including T cell activation, leukocyte activation, adhesion and migration, and immune response regulation. Within the CC category, DEGs were significantly enriched in pathways related to intracellular components, cytoplasmic vesicles, secretory granule membranes, and other related component pathways. Within the MF category, DEGs were significantly enriched in signal transduction and other functional pathways, such as amide binding, as well as immune receptor activity (Figure 3a). KEGG pathway analysis revealed significant enrichment of DEGs in pathways related to tuberculosis, phagosomes, and the chemokine signaling pathway (Figure 3b). These findings indicated that these DEGs were significantly enriched in immune and inflammatory pathways, including immune cell activation and migration, vesicle trafficking, and chemokine signaling. These findings suggest that immune and inflammatory reactions are potential key factors in the occurrence and development of atherosclerosis.

Figure 3(a) Bar chart of GO enrichment analysis of differentially expressed genes related to atherosclerosis, including BP, CC, and MF pathways. (b) Bubble plot of KEGG enrichment analysis of differentially expressed genes related to atherosclerosis.

**Figure:**
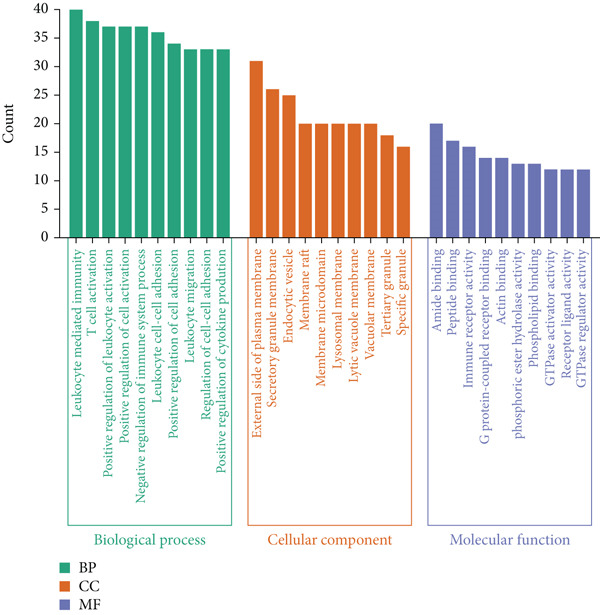
(a)

**Figure:**
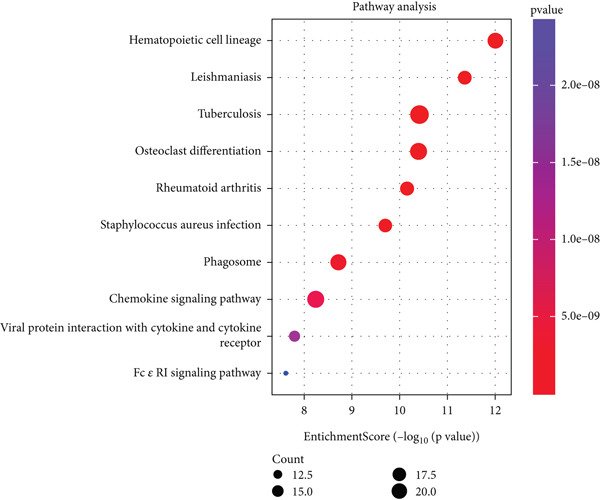
(b)

### 3.3. WGCNA Analysis and Screening of Hub Genes

To further identify key genes in atherosclerosis, WGCNA was performed on 17,547 genes from a new dataset. First, the bottom 50% of genes were deleted based on their median absolute deviation (MAD). For matrix definition within the WGCNA, a soft threshold of 8 was adopted (Figure 4a,b), ensuring a scale‐free *R* ^2^ = 0.86 to fit the expression of genes linked to scale‐free networks. After raising the adjacency matrix to the power of 8, it was converted into a topological overlap matrix (TOM). The TOM matrix was used to evaluate the network connectivity of each gene, defined as the sum of its adjacencies with all other genes in the network. The corresponding dissimilarity (1‐TOM) was computed. To group genes with comparable expression patterns into distinct modules, a minimum module size of 30 genes was applied as a threshold in the gene dendrogram. A hierarchical clustering analysis, using the average linkage algorithm, was performed on the dissimilarity data obtained from the TOM. The sensitivity for this process was set to 3. For further module analysis, module eigengenes were correlated with phenotypic traits. Modules were then merged based on a dissimilarity threshold of 0.25 (1‐TOM), yielding a final set of 22 coexpressed modules (Figure 4c). Notably, the gray module represented a collection of genes that were not assigned to any defined module. Module–trait analysis demonstrated the connections between different colored modules and atherosclerosis. A correlation evaluation was conducted to assess the correlation of each feature gene module with atherosclerosis (Figure 4d). Based on this analysis, we selected the three modules with the highest positive correlations (turquoise, midnight blue, and orange) and the module with the highest negative correlation (salmon). Subsequently, 1400 genes were extracted from these four modules. The intersection of DEGs, WGCNA target genes, and 794 ARGs was determined using a Venn diagram (Figure 4e). The resulting 13 overlapping genes represented key candidate genes with important physiological regulatory functions.

Figure 4(a) Scale independence of WGCNA, *β* = 8, 0.86. (b) Average connectivity of WGCNA, *β* = 8, 51.25. (c) Clustering of 22 module feature vectors in WGCNA. (d) Heat map of correlation between module and phenotype in WGCNA. (e) Venn diagram for intersection genes.

**Figure:**
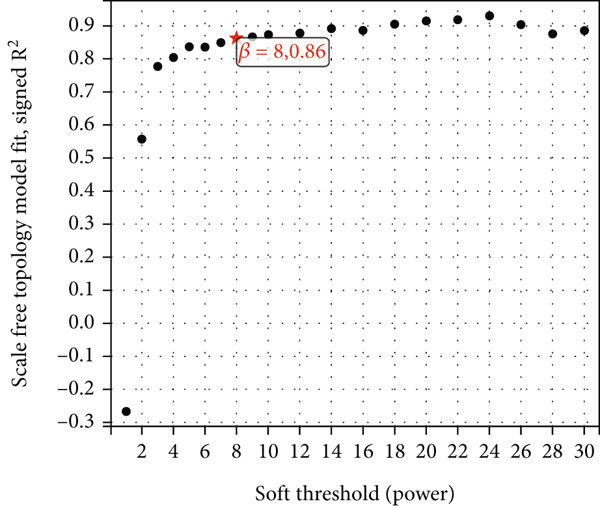
(a)

**Figure:**
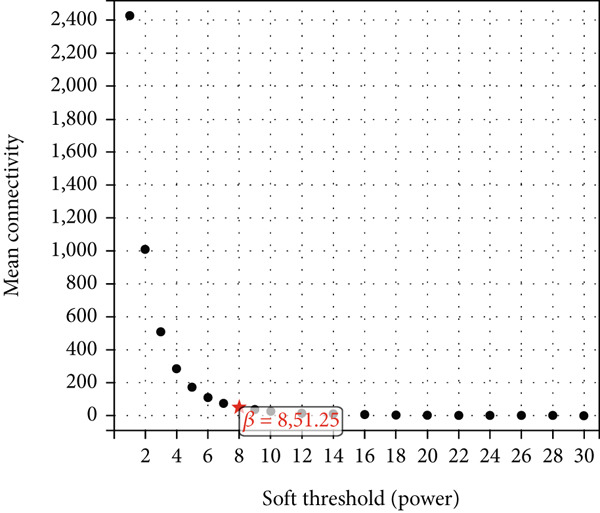
(b)

**Figure:**
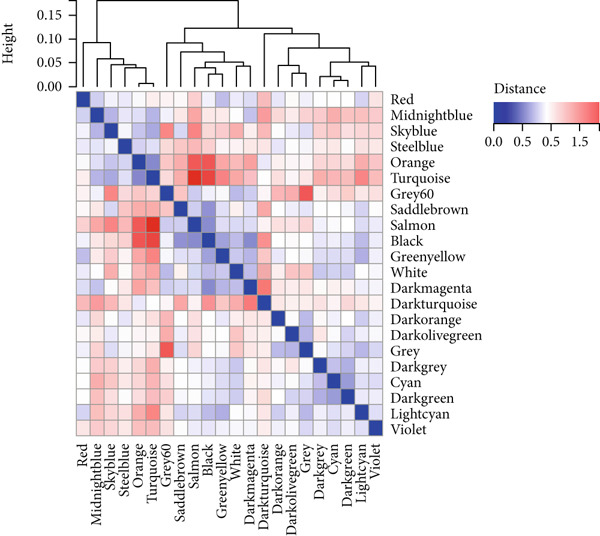
(c)

**Figure:**
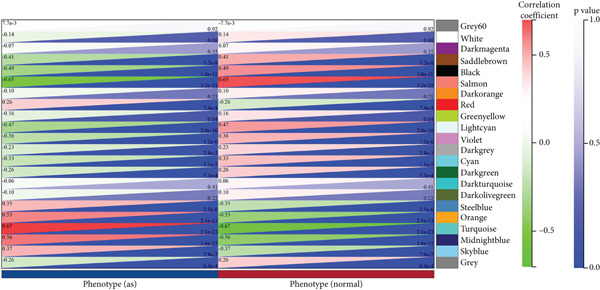
(d)

**Figure:**
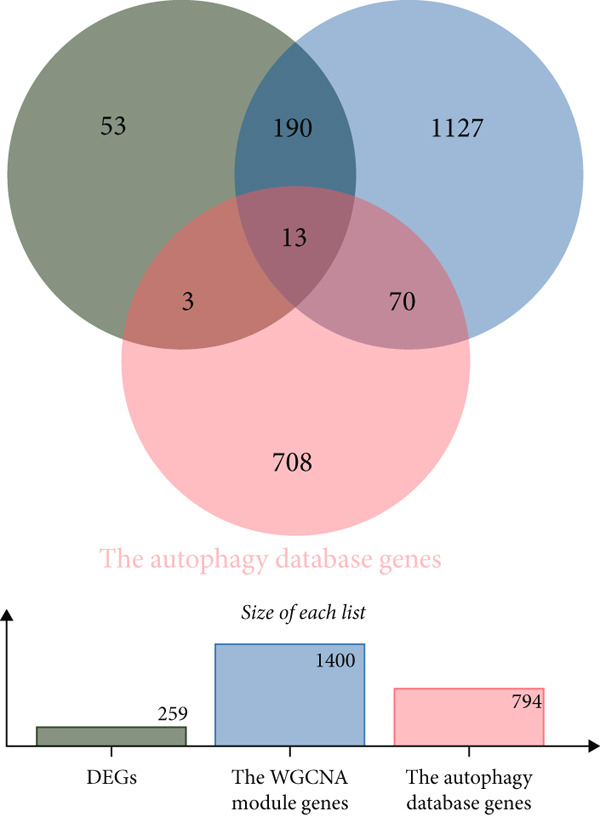
(e)

### 3.4. Application of Machine Learning Algorithms for Key Gene Screening

To further identify key genes from the overlapping genes, we employed four machine learning algorithms: ridge regression, LASSO logistic regression, random forest, and XGBoost. Gene importance was evaluated using both the Gini coefficient and accuracy measures within the random forest algorithm. Subsequently, the Top 9 genes ranked by Gini coefficient were selected (Figure 5a,b). LASSO regression analysis identified 10 key genes (Figure 5c,d). Ridge regression analysis selected 13 key genes (Figure 5e,f). In the XGBoost algorithm, the nine variables exhibiting the greatest relative importance were chosen as key genes (Figure 5g). The overlapping genes identified by the four different algorithms were extracted using a Venn diagram. Finally, seven signature genes were identified (Figure 5h): HSPB8, MYOCD, TLR7, heme oxygenase 1 gene (HMOX1), cardiac ryanodine receptor 2 (RYR2), NCF1, and TBC1D10C.

Figure 5(a) Gini coefficient of each gene in random forest algorithm. (b) Accuracy of each gene in random forest algorithm. (c) Variable screening of LASSO regression analysis. (d) Line graph of feature change vector in LASSO regression analysis. (e) Variable screening of ridge regression analysis. (f) Line graph of feature change vector in ridge regression analysis. (g) Importance of each gene in XGBoost algorithm. (h) Venn diagram for intersection genes of four algorithms.

**Figure:**
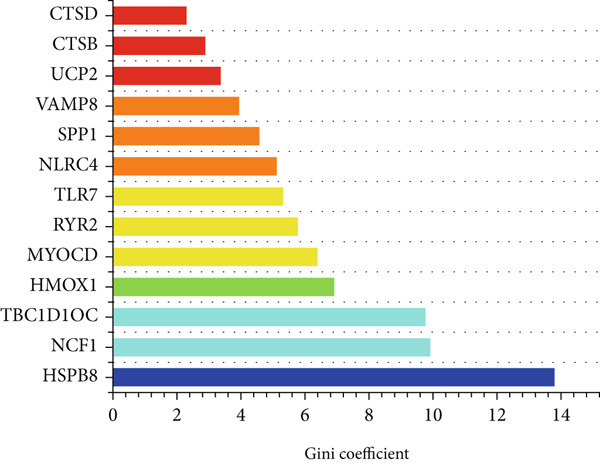
(a)

**Figure:**
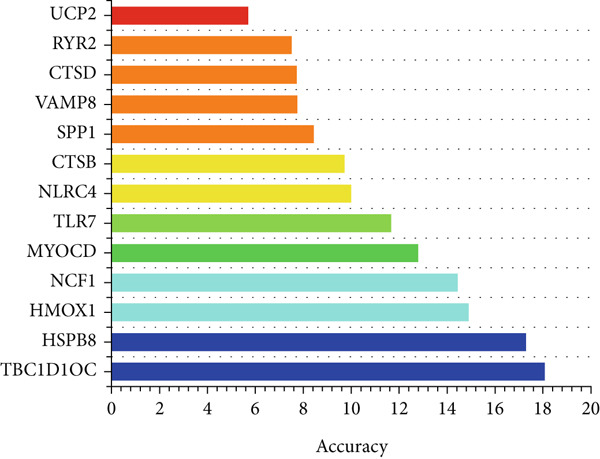
(b)

**Figure:**
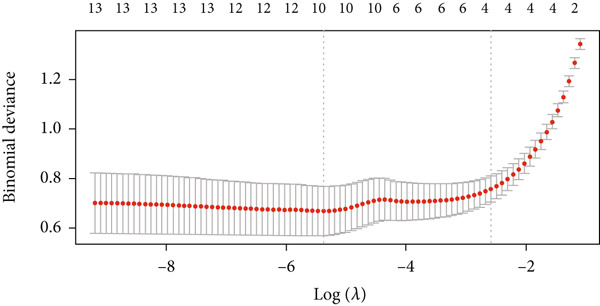
(c)

**Figure:**
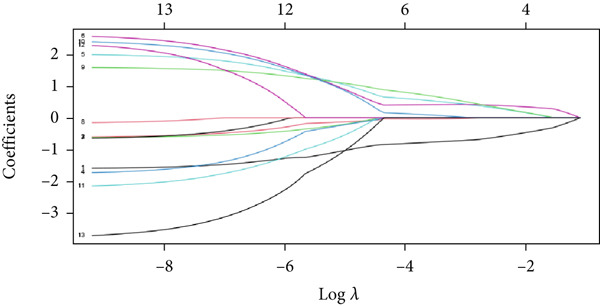
(d)

**Figure:**
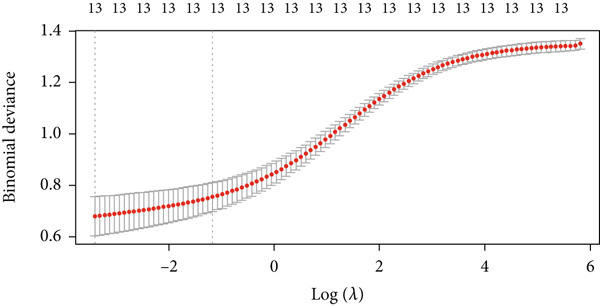
(e)

**Figure:**
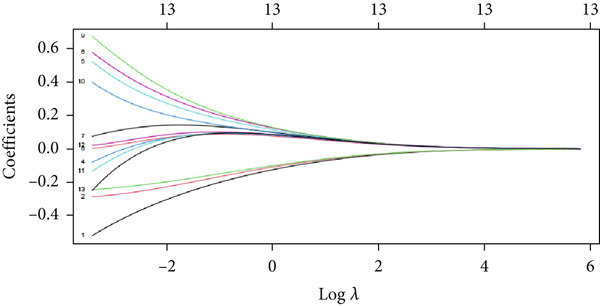
(f)

**Figure:**
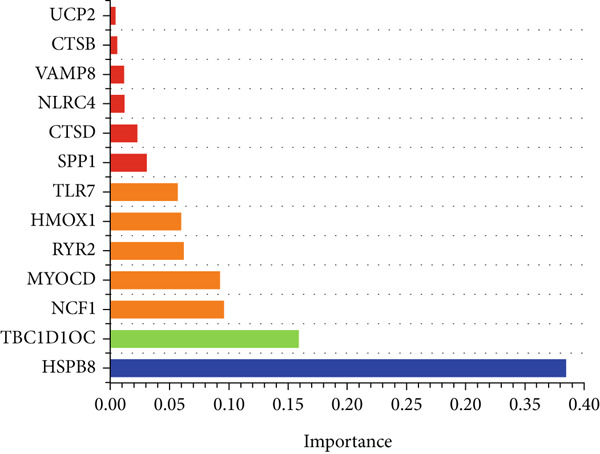
(g)

**Figure:**
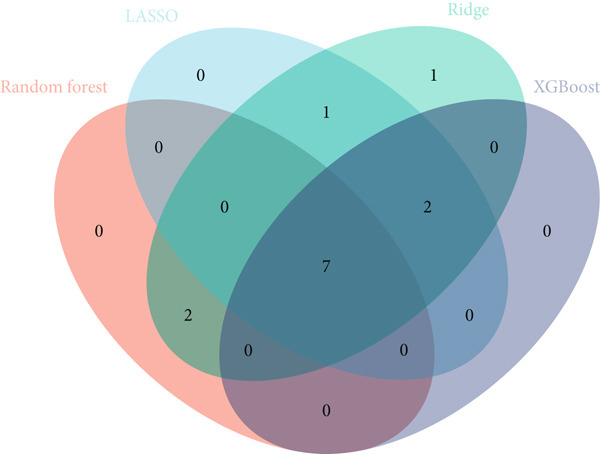
(h)

### 3.5. Construction of Autophagy–Atherosclerosis Prediction Model

In order to explore the diagnostic accuracy of these seven genes for autophagy‐related atherosclerosis, we generated ROC curves (Figure 6a) and selected genes with area under curve (AUC) value > 0.6 as diagnostic markers. Notably, HSPB8 exhibited the highest AUC value (0.912) among the seven genes. The AUC values for TLR7, RYR2, HSPB8, HMOX1, NCF1, and TBC1D10C were 0.853, 0.862, 0.863, 0.883, 0.901, and 0.901, respectively. These findings indicated that these seven genes could be used as diagnostic markers. In the external validation dataset GSE57691, we also constructed ROC curves for seven genes to verify their diagnostic sensitivity and specificity for atherosclerosis (Figure 6b). The ROC values of the other six genes (excluding TLR7) were all greater than 0.6, indicating their potential as diagnostic biomarkers for AS. In addition, the prediction model for atherosclerosis was established using logistic regression based on the expression values of these six genes. The accuracy, sensitivity, specificity, positive predictive value, and negative predictive value were 73.68%, 88.89%, 60%, 66.67%, and 85.71%, respectively (Figure 6c), indicating higher accuracy in the validation set. The ROC value of the prediction model was 0.833, demonstrating high diagnostic feasibility (Figure 6d).

Figure 6(a) ROC curve analysis of hub genes in the training set. (b) ROC curve analysis of hub genes in the validation set. (c) Various indicators of the prediction model. (d) ROC curve analysis of the diagnostic model.

**Figure:**
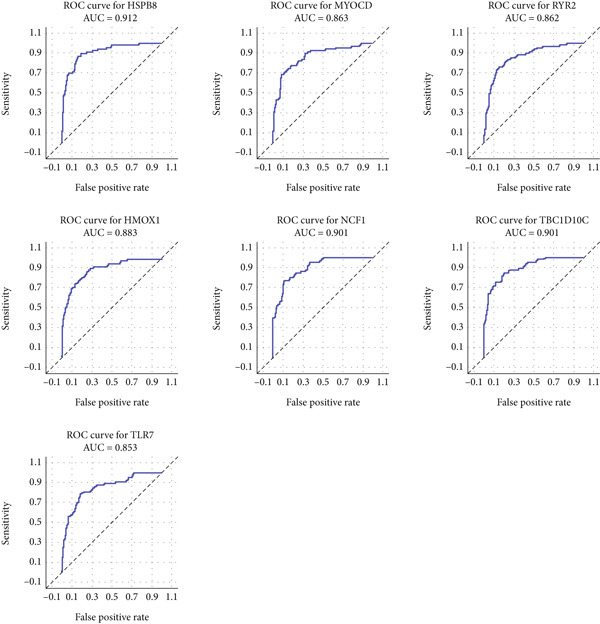
(a)

**Figure:**
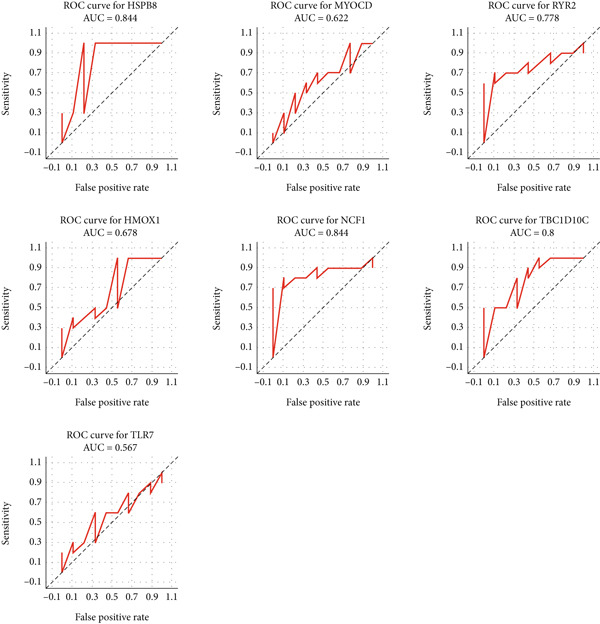
(b)

**Figure:**
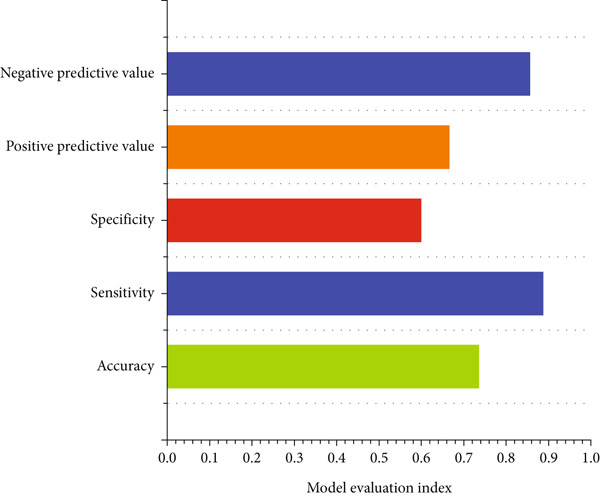
(c)

**Figure:**
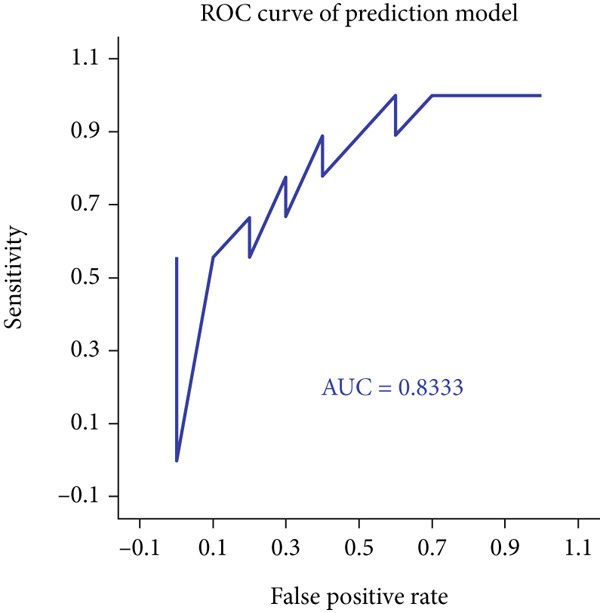
(d)

### 3.6. Identification of Key Gene‐Related Clusters in Atherosclerosis

Consensus clustering was utilized to classify AS samples according to the expression data of 13 key genes, allowing us to investigate autophagy expression patterns in AS. The results, when divided into C1 and C2 groups, exhibited the most stable patterns (Figures 7a, 7b, and 7c). PCA indicated a pronounced difference between the two groups (Figure 7d). To understand the molecular distinctions between the clusters, the expression patterns of 13 key genes were compared between C1 and C2. The results showed that HMOX1, NLRC4, NCF1, TBC1D10C, TLR7, SPP1, CTSD, UCP2, CTSB, and VAMP8 expressions were elevated in C1, while the expression levels of RYR2, HSPB8, and MYOCD were markedly elevated in C2 (Figure 7e). The variation in immune infiltration was also evaluated between the two groups. C1 was enriched in activated memory T cells, *γδ* T cells, regulatory T cells, and M0 macrophages. In contrast, C2 showed higher levels of plasma cells, CD8 T cells, resting memory CD4 T cells, activated NK cells, monocytes, M2 macrophages, and resting mast cells (Figure 7f).

Figure 7(a) CDF values. (b) Changes in CDF values and corresponding areas under the curve, *k* = 2–6. (c) Consistency clustering matrix, *k* = 2. (d) PCA diagram of clusters C1 and C2. (e) Heat map of expression of 13 genes in clusters C1 and C2. (f) Analysis of immune cell infiltration levels in clusters C1 and C2 using CIBERSORT algorithm. (g) Analysis of immune infiltration in the merged dataset AS and normal samples using CIBERSORT algorithm. (h) TF‐miRNA‐mRNA regulatory network. Notes: “‐” indicates not significant, “.” indicates *p* less than 0.1, “∗” indicates *p* < 0.05, “∗∗” indicates *p* < 0.01, and “∗∗∗” indicates *p* < 0.001. Red represents mRNA, green represents TF, and yellow represents miRNA.

**Figure:**
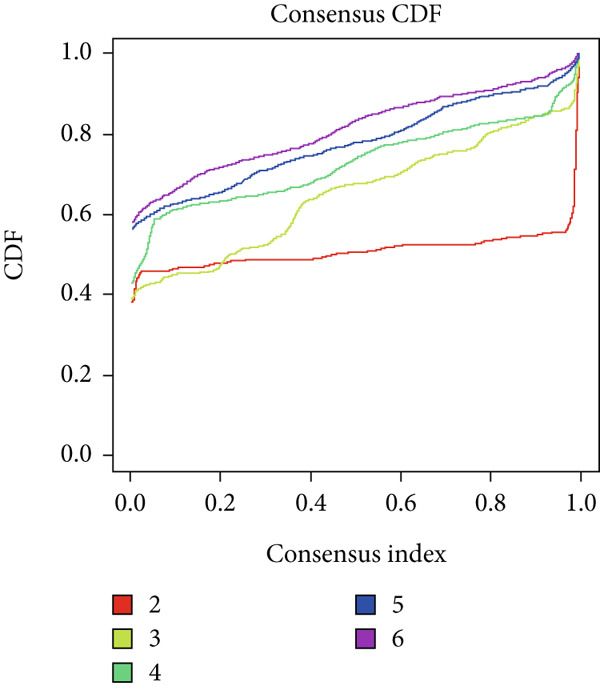
(a)

**Figure:**
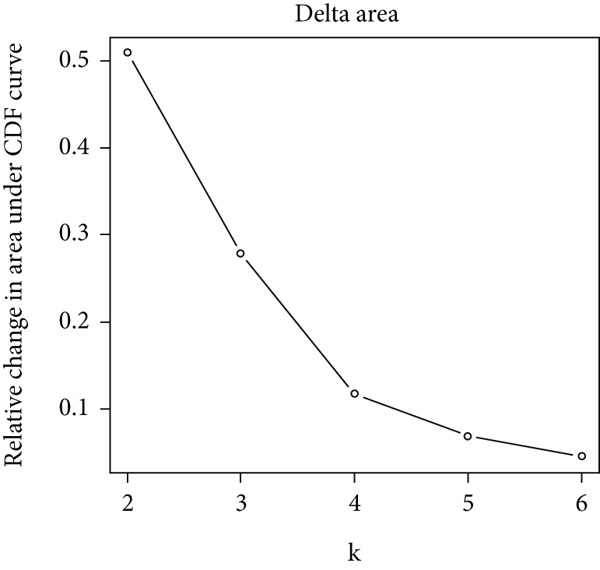
(b)

**Figure:**
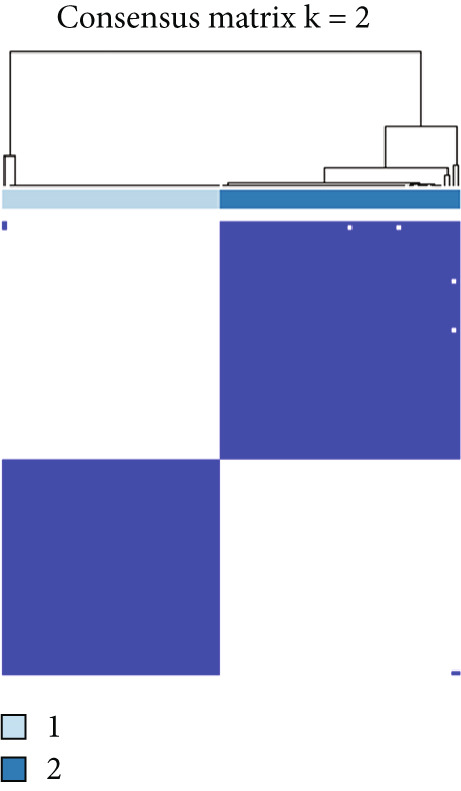
(c)

**Figure:**
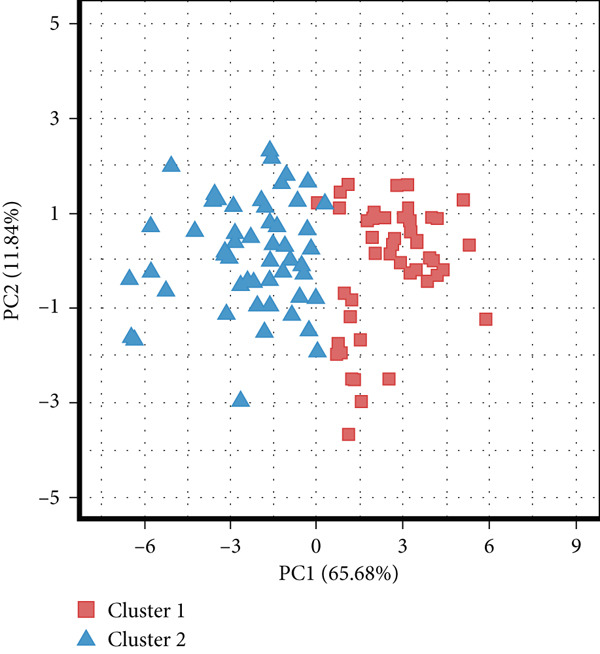
(d)

**Figure:**
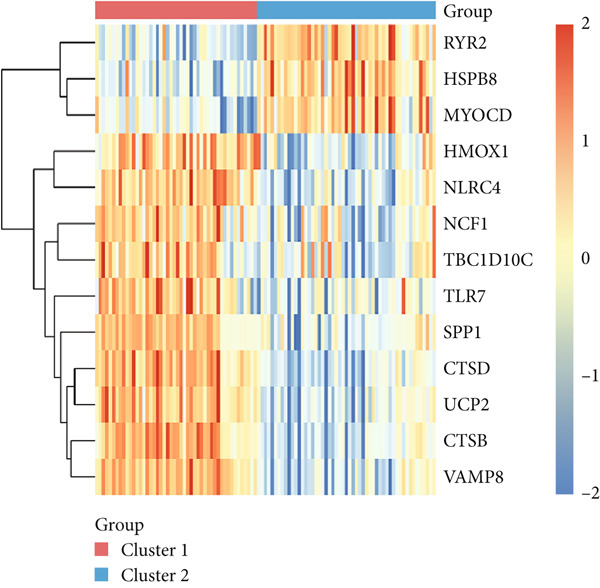
(e)

**Figure:**
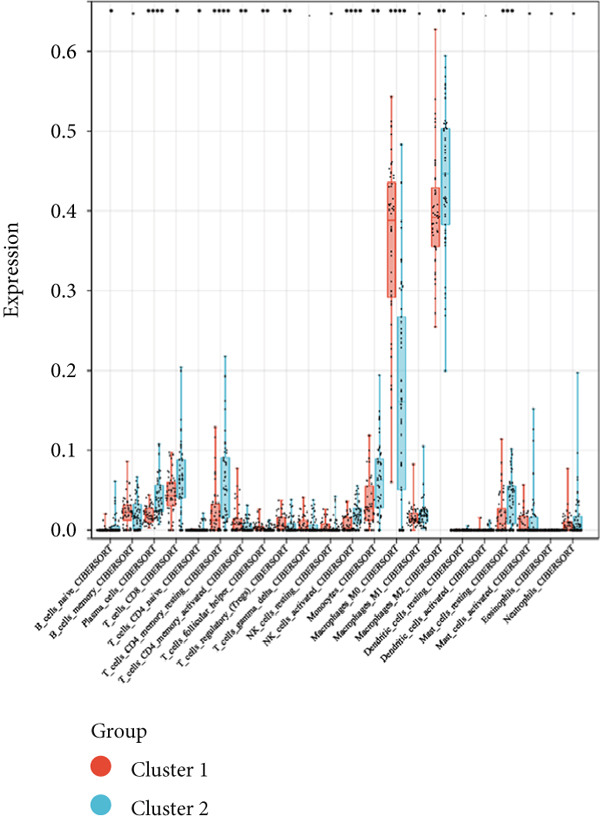
(f)

**Figure:**
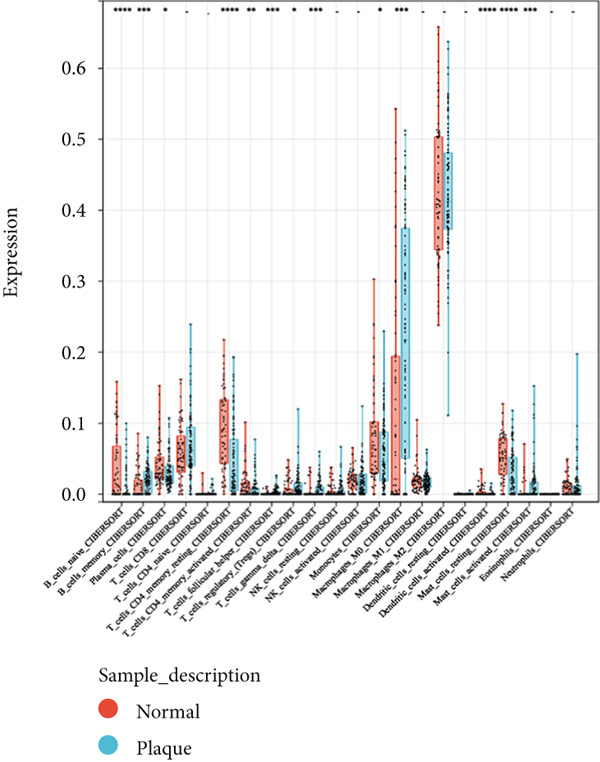
(g)

**Figure:**
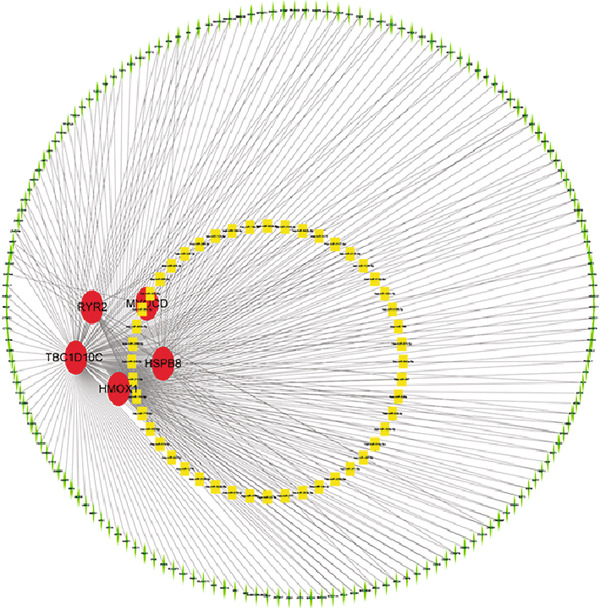
(h)

### 3.7. Immune Infiltration Analysis

Given the enrichment of key diagnostic genes for atherosclerosis in immune‐related pathways, an immune infiltration analysis was executed to gain a deeper understanding of the immune system’s role in the development of AS. In comparison to the control group, the atherosclerosis group demonstrated reduced levels of naïve B cells, plasma cells, CD4+ memory resting T cells, CD4+ memory activated T cells, monocytes, resting mast cells, and activated dendritic cells. Conversely, the atherosclerosis group exhibited higher levels of memory B cells, regulatory T cells, CD8+ T cells, gamma delta T cells (*γδ* T cells), M0 macrophages, and activated mast cells (Figure 7g). Overall, the immune infiltration was more pronounced in the atherosclerotic plaque group, and the variable immune cell infiltration observed in atherosclerotic patients indicated a possible therapeutic target.

### 3.8. Construction of TF‐miRNA‐mRNA Regulatory Network

To gain further insight into the roles of the identified hub genes within a network context, miRNAs possibly bound by hub genes and transcription factors of hub genes were determined using public databases. By utilizing the starBase database and its six recorded datasets, miRNAs predicted to target the hub genes were identified. We selected results that appeared in at least two datasets and had at least two CLIP‐seq experiments and constructed a hub gene–miRNA network, resulting in 56 interaction pairs (Table S3). Subsequently, 253 related TFs were obtained from the hTFtarget database (Table S4). A regulatory network was constructed based on their interaction relationships (Figure 7h). This network was essential for comprehending the intricate hierarchy of gene regulation within cells, the mechanisms of gene activation and repression, the role of miRNAs in fine‐tuning this regulation, and the complex equilibrium that sustains normal cellular function or contributes to disease when disrupted.

### 3.9. Verification of Hub Gene Expression in Macrophage‐Derived Foam Cells

The real‐time PCR (RT‐PCR) technique was employed to examine the mRNA expression profiles of seven hub genes in an atherosclerosis model. Humanized macrophages served as the control group, while humanized foam cells constituted the experimental group. RT‐PCR analysis demonstrated that mRNA expression data for the six hub genes in control and experimental groups were consistent with the predicted results (Figures 8a, 8b, 8c, 8d, 8e, and 8f).

Figure 8(a) Comparison of mRNA expression levels of HSPB8 between the control group and experimental group. (b) Comparison of mRNA expression levels of MYOCD between the control group and experimental group. (c) Comparison of mRNA expression levels of RYR2 between the control group and experimental group. (d) Comparison of mRNA expression levels of HMOX1 between the control group and experimental group. (e) Comparison of mRNA expression levels of TBC1D10C between the control group and experimental group. (f) Comparison of mRNA expression levels of NCF1 between the control group and experimental group. Notes: “ns” indicates not significant, “∗” indicates *p* < 0.05, “∗∗” indicates *p* < 0.01, and “∗∗∗” indicates *p* < 0.001.

**Figure:**
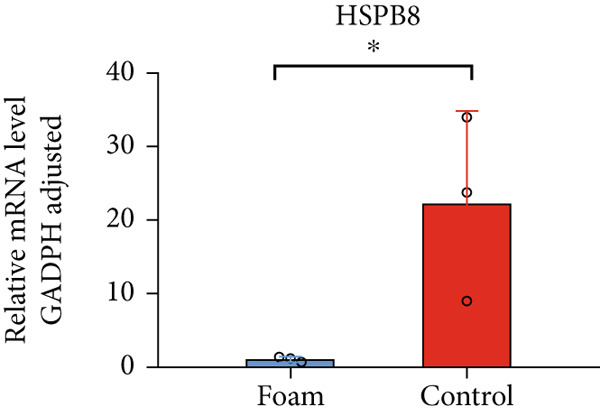
(a)

**Figure:**
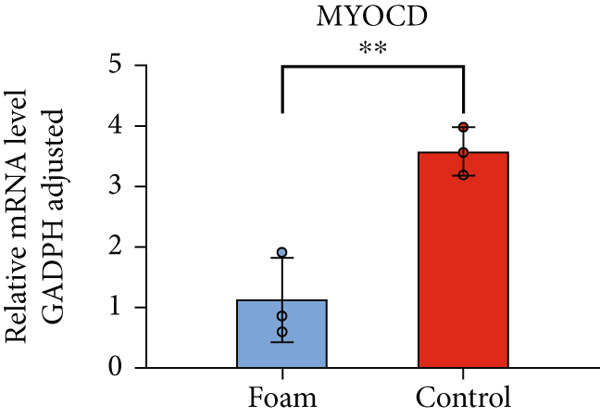
(b)

**Figure:**
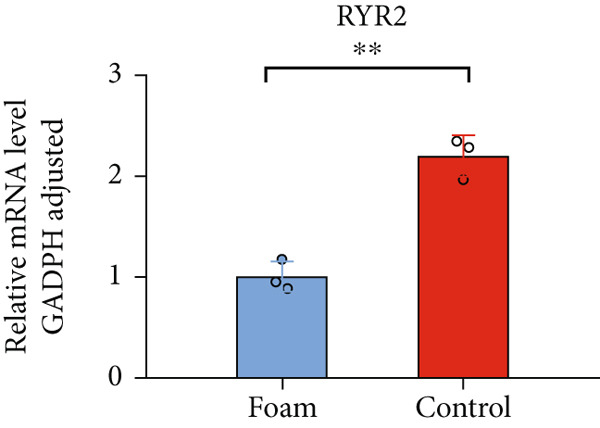
(c)

**Figure:**
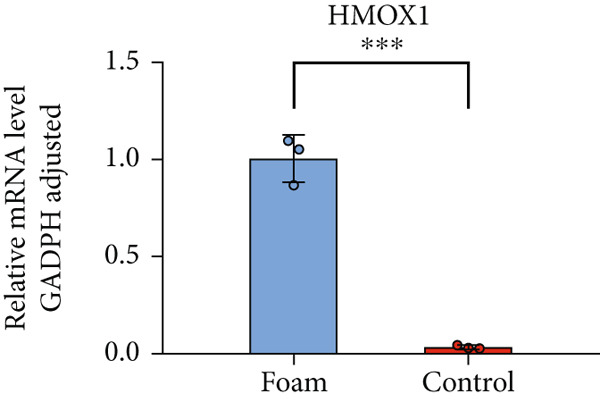
(d)

**Figure:**
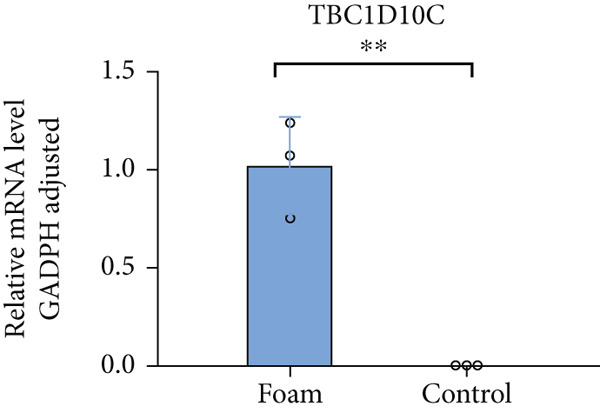
(e)

**Figure:**
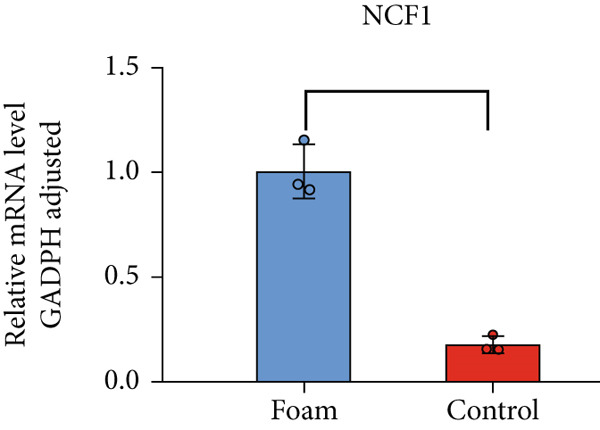
(f)

## 4. Discussion

With the decline in the incidence of infectious diseases, atherosclerosis has become a primary health challenge for humans (1). Extensive research indicates that autophagy is essential for cardiovascular homeostasis, and its dysfunction contributes to atherosclerotic plaque formation (2). Therefore, it is of significant importance to identify autophagy‐regulating genes that contribute to the advancement and evolution of atherosclerotic plaques.

By analyzing two atherosclerotic plaque datasets from the GEO database through bioinformatics techniques, we ultimately identified six key genes involved in both atherosclerosis and autophagy regulation utilizing four machine learning algorithms. The expression of these pivotal genes in atherosclerotic plaques was additionally validated through RT‐PCR findings. These six genes represent potential new targets for the treatment of atherosclerosis, and their functions are explored in detail below.

TBC1D10C, recognized as carabin, functions as a Ras‐binding protein that exerts a negative regulatory role in T‐cell signaling. Its C‐terminal RAP domain plays a crucial role in inhibiting the Ras pathway and calcineurin, thereby crucially suppressing the activation of CD4+ T and B cells [23, 24]. Autophagy in T cells is dependent on the activation by TCR signals [25], and the inhibitory action of TBC1D10C might suppress this process [26], which could be one of the mechanisms underlying the function of the autophagy inhibitor TBC1D10C. In human atherosclerotic plaques, TBC1D10C expression is notably elevated, and the dysregulation of autophagy is intimately linked to the progression of atherosclerotic plaques. Given that TBC1D10C functions as an autophagy inhibitor, it can be deduced that TBC1D10C contributes to the risk factors associated with atherosclerosis. Currently, the functional mechanisms of TBC1D10C in atherosclerosis are not fully understood, and further exploration is needed to understand its involvement in the onset and advancement of atherosclerosis.

HMOX1 demonstrates antioxidant, anti‐inflammatory, and antiapoptotic roles and is linked to the formation of atherosclerotic lesions [27]. In addition, HMOX1 plays a significant role in autophagy signaling. It has been demonstrated that LPS‐induced activation of TLR4 in macrophages leads to increased HMOX1 expression, subsequently promoting autophagy, which is essential for macrophages to manage inflammatory processes [28]. The research findings revealed that HMOX1 is prominently expressed in atherosclerotic plaques and acts as an autophagy regulator, holding a pivotal modulatory role in atherosclerosis. Prior research has shown that inhibition of HMOX1 exacerbates atherosclerotic lesions, while induction of HMOX1 alleviates these lesions, providing evidence for the antiatherosclerotic role of HMOX1 [29]. This protective mechanism is likely due to Nrf2/HMOX1 pathway‐induced autophagy [30].

The cytosolic subunit p47phox of NADPH oxidase, encoded by the NCF1 gene, undergoes phosphorylation upon stimulation of phagocytes. This phosphorylation event triggers a conformational shift in p47phox, resulting in the activation of the NADPH oxidase complex [31]. NADPH oxidase significantly contributes to reactive oxygen species (ROS) production. In atherosclerotic lesions, ROS may play a significant role in disease progression by oxidatively altering low‐density lipoprotein (LDL), deactivating nitric oxide (NO), and influencing redox‐sensitive signaling pathways [32]. Notably, NADPH oxidase deficiency has been shown to attenuate atherosclerosis in apoE(−/−) mice [33]. The observed increase in NCF1 expression within atherosclerotic plaques may be associated with augmented ROS production in these lesions. Furthermore, NCF1 exhibits a close relationship with autophagy. Specifically, Nox2‐derived superoxide has been shown to impair lysosomal function and affect enzymatic activity within the lysosome. Conversely, deletion of p47phox restores lysosomal acidification and subsequently recovers autophagic flux [34]. These findings suggest a novel mechanism by which NCF1 may regulate the progression of atherosclerosis.

HSPB8 plays a role in recognizing misfolded proteins within chaperone‐mediated selective autophagy [35]. At present, investigations into the function of HSPB8 in atherosclerosis are scarce, and the underlying mechanisms through which HSPB8 may impact atherosclerosis are yet to be determined.

RYR2 modulates calcium signaling, which in turn influences autophagy [36]. The involvement of RYR2 in the progression of atherosclerotic plaques is not yet clearly understood. Our study demonstrates markedly decreased expression of RYR2 in atherosclerotic plaques.

MYOCD is known to play a crucial role in cardiovascular disease. For example, MYOCD deficiency exacerbates atherosclerosis by downregulating ABCA1‐dependent cholesterol efflux in VSMCs [37]. Recent research has shown that MYOCD can suppress autophagy by promoting the transcription of miR‐30a [38], suggesting that MYOCD might influence the regulation of atherosclerosis by modulating autophagic processes.

Four machine learning models were constructed to identify seven autophagy‐regulating genes. Six genes demonstrated high AUC scores in both the training and verification datasets. The results of the prediction model showed that these six genes exhibited strong diagnostic potential as biomarkers. Atherosclerosis is considered a chronic inflammatory condition, with its advancement closely tied to the presence and activity of inflammatory cells [39]. Hence, we conducted a comparison of immune infiltration disparities between Clusters C1 and C2. It was evident that C2 displayed a notably greater prevalence of immune response cells in comparison to C1. To delve deeper into the potential mechanisms of these six autophagy‐regulating genes, we investigated their relationship with immune cells. Interestingly, these genes exhibited a strong correlation with M0 macrophages. Subsequently, we validated our hypothesis through quantitative real‐time PCR (qRT‐PCR) assays in macrophages and foam cells. The results were consistent with our expectations. In conclusion, autophagy‐regulating genes likely contribute to atherosclerosis pathogenesis and progression by affecting macrophages and the immune microenvironment.

Through bioinformatics analysis, our research has determined a group of genes whose effects on atherosclerosis are mediated by autophagy. These genes are promising diagnostic biomarkers for the condition, as well as therapeutic targets for interventions aimed at delaying atherosclerotic progression and stabilizing plaques. Nonetheless, our study has its limitations. Due to the lack of public databases related to atherosclerosis, we selected only two datasets, and the sample size was relatively small. Moreover, additional experiments are needed to clarify the mechanisms through which these genes genuinely influence atherosclerotic plaques.

## 5. Conclusion

This study has identified six genes (HSPB8, MYOCD, HMOX1, RYR2, NCF1, and TBC1D10C) associated with both atherosclerotic progression and autophagy regulation through integrative analysis. qRT‐PCR has been utilized to experimentally validate these genes, providing a reference for the mechanistic study and molecular therapy of atherosclerotic plaque. These six genes exhibit favorable diagnostic performance.

## Disclosure

All authors commented on previous versions of the manuscript. All authors read and approved the final manuscript.

## Conflicts of Interest

The authors declare no conflicts of interest.

## Author Contributions

**Ao Yin:** conceptualization, methodology, software. **Xingyu Fu:** data curation, writing – original draft preparation. **Xinxin Liu:** visualization, investigation. **Min Li:** supervision. **Xiaochen Yu:** software, validation. **Xiuru Guan:** writing – reviewing and editing.

## Funding

No funding was received for this manuscript.

## Supporting Information

Additional supporting information can be found online in the Supporting Information section.

## Supporting information


**Supporting Information 1** Table S1: List of autophagy‐related genes.

**Supporting Information 2** Table S2: Primer information.

**Supporting Information 3** Table S3: mRNA‐miRAN network.

**Supporting Information 4** Table S4: mRNA‐TF network.

## Data Availability

The datasets (GSE43292, GSE100927 and GSE57691) that support the findings of this study are available from the GEO database (http://www.ncbi.nlm.nih.gov/geo).
